# Patrolling Mechanics of Non-Classical Monocytes in Vascular Inflammation

**DOI:** 10.3389/fcvm.2017.00080

**Published:** 2017-12-19

**Authors:** Konrad Buscher, Paola Marcovecchio, Catherine C. Hedrick, Klaus Ley

**Affiliations:** ^1^Division of Inflammation Biology, La Jolla Institute for Allergy and Immunology, La Jolla, CA, United States; ^2^Department of Internal Medicine, Nephrology and Rheumatology, University Hospital Münster, Münster, Germany

**Keywords:** monocytes, patrolling, arteriosclerosis, arteries, microcirculation, venules

## Abstract

Non-classical monocytes have emerged as the preeminent vascular housekeepers. Continuous intravascular screening is enabled by slow patrolling on the endothelium and allows a rapid response to local perturbations. Intravital imaging has been crucial to elucidate the molecular mechanisms and migratory phenotype of patrolling. In this review, we discuss technical requirements of intravital microscopy such as imaging modalities, labeling strategies, and data analysis. We further focus on patrolling kinetics and adhesion receptors in different organs and vascular beds including arteries during homeostasis and vascular inflammation and define pertinent questions in the field.

## Introduction

Monocytes have been implicated in many inflammatory diseases (1, 2). They are composed of at least two murine monocyte populations with distinct functional and molecular properties (3). Ly6C^+^ CCR2^+^ CX3CR1^−^ classical monocytes are abundant in the blood and in several non-inflamed organs (spleen, lung, liver, and brain), and readily extravasate to many inflammatory sites (3). In contrast, Ly6C^−^ CCR2^−^ CX3CR1^+^ non-classical monocytes predominantly remain in the vascular system (3) and engage in long-term migration along the endothelium with or against the flow, a process termed patrolling (4). Transcriptomic and functional comparison suggests that CD14^−^CD16^+^ monocytes are the human counterpart to patrolling monocytes (=non-classical monocytes; both terms are interchangeable) in mice (5–7). Non-classical monocytes can derive from classical monocytes and have a lifespan of several days in humans (8) and mice (9, 10). Activated endothelial cells attract patrolling monocytes for scavenging and neutrophil-mediated necrosis (11). Similarly, endothelium of the pulmonary circulation of tumor-bearing mice attracts patrollers that subsequently orchestrate an antitumor response by recruiting NK cells (12). Viruses or nucleic acids induce a TLR7-mediated response in patrollers that results in the production of TNF-α, IL-1β, and CCL3 (5). Non-classical monocytes often exert anti-inflammatory and prohomeostatic effects (5, 11, 13). However, they can also have pro-inflammatory functions depending on the disease-specific context (13–15). Further insights into patrolling mechanisms will be critical to understand and therapeutically target the leukocyte response in cardiovascular disease (16).

*In vitro* culture of endothelial cell layers has been instrumental in understanding monocyte behavior (17). However, the full repertoire of adhesion molecules and signaling cues underlying effective patrolling still remains obscure. As a result, patrolling cannot be studied *in vitro* using purified ligands as immobilized substrates. Moreover, data suggest that the molecular and migratory phenotype differs between vessel compartments [arteries vs. venules (18)] and tissues [ear dermis venules (19) vs. kidney cortex circulation (11) vs. mesenteric venules (19, 20)]. Therefore, intravital microscopy in anesthetized mice is paramount to the study of patrolling monocytes. It provides an *in situ* characterization of migration patterns, endothelial interactions, and the local orchestration of a dynamic leukocyte response. This review elaborates on the imaging technology, labeling strategies, migration phenotypes, and molecular requirements of patrolling monocytes throughout the circulation in healthy and inflammatory conditions.

## Labeling Modalities

Although CX3CR1-GFP and Nr4a1-GFP mice are widely used to image patrollers, there are currently no reporter mouse strains with highly specific endogenous markers. Alternative approaches to imaging patrolling monocytes *in vivo* are feasible but require a number of experimental considerations.

**Figure 1:**
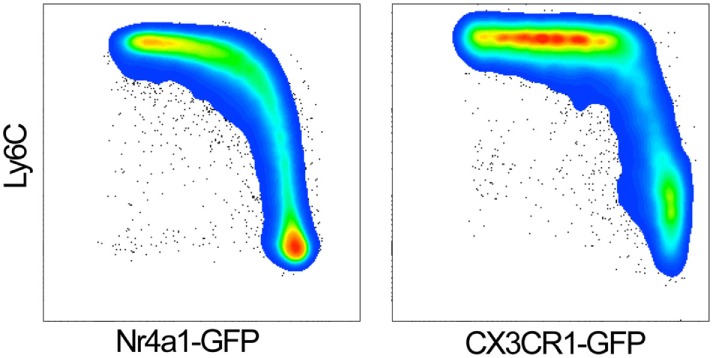
GFP expression in classical (Ly6C^high^) and non-classical monocytes (Ly6C^low^) of Nr4a1-GFP (left panel) and CX3CR1-GFP^+/−^ mice (right panel). Gated on single live CD45^+^, CD115^+^ CD11b^+^ monocytes.

Specificity (true negative rate) and sensitivity (true positive rate) determine the value of any labeling strategy. Many reporter mice lack sensitivity, i.e., many non-targeted cells are also labeled. The CX3CR1-GFP mouse is widely used for studies of the mononuclear phagocyte system. The CX3CR1 locus had been replaced with an eGFP construct (knock-in), resulting in cytosolic GFP fluorescence (21). In heterozygotes (CX3CR1-GFP^+/−^), several myeloid lineages are GFP^+^ including monocytes, dendritic cells, tissue-resident macrophages, brain microglia, and subsets of NK and T cells (21). Monoallelic expression of CX3CR1 seems sufficient for adequate chemokine receptor function, although this has not been tested rigorously, and alterations have been reported (22). Homozygous GFP expression (CX3CR1-GFP^+/+^) results in a CX3CR1 knockout. Comparing CX3CR1-GFP^−/+^ with CX3CR1-GFP^+/+^ in littermates is useful for understanding the functional role of CX3CR1 in monocytes (23). Due to the long half-life of unmodified eGFP, the eGFP signal does not correlate well with the endogenous CX3CR1 expression (24). Non-classical and classical monocytes show a high and intermediate GFP expression, respectively (Figure 1). With high sensitivity photomultiplier tubes in modern microscopes, both monocyte subsets are detectable. Thorough controls are required to ensure the sensitivity of the GFP signal, such as complementary flow cytometry data (e.g., to show that recorded CX3CR1-GFP^+^ cells in a specific disease model are indeed patrolling monocytes and not classical monocytes or other leukocyte lineages).

To overcome the issue of CX3CR1-GFP expression in other blood leukocytes, these mice were crossed with IL2RG^−/−^ Rag2^−/−^ knockout strains (19). Here, monocytes remain the only GFP^+^ population in the blood (19). However, the global gamma chain deficiency and lack of T cells in Rag2^−/−^ mice severely alters systemic immunity (25), limiting the applicability of this mouse model.

Another common reporter for non-classical monocytes is the transgenic Nr4a1-GFP (Nur77-GFP) mouse. This mouse was originally generated for the study of TCR activation (26). A GFP-Cre fusion protein was inserted at the start codon of Nr4a1, and fluorescence is induced by antigen stimulation. It was later discovered that Nr4a1 is mandatory for the development of non-classical monocytes in the bone marrow (27). Nr4a1-GFP reporter mice show a strong GFP signal in non-classical monocytes, whereas classical monocytes are low (27). The GFP intensity of the two monocyte subsets is about one magnitude further apart than in CX3CR1-GFP mice (Figure 1). This suggests that GFP^high^ and GFP^low^ discrimination in Nr4a1-GFP mice may be superior to CX3CR1-GFP mice for intravital microscopy.

*In vivo* labeling by antibodies or dyes (28) is an alternative or complementary approach to visualize patrolling monocytes. It is critical that azide is removed from commercially available products, which can be done by using microdialysis or spin columns. Some companies provide no azide (NA)/low endotoxin (LE) antibodies for *in vivo* applications. Depending on the abundance of the target, 1–5 µg suffice to image monocytes. Caveats include adverse effects of antibody binding, such as function blocking, receptor dimerization, internalization, or presentation of the Fc portion to Fc receptors on monocytes, endothelial, or other cells. All these can lead to unwanted activation (or inhibition) of downstream effects. The use of F_ab_ fragments circumvents the latter problem, but does not address internalization or function-blocking issues (29). As an example, the anti-CD11b antibody clone M1/70 is commonly used to tag myeloid cells *in vivo* (11), but its function-blocking effect may alter patrolling kinetics. This was shown in untreated mesentery venules (20) and in TLR7 agonist R848-treated venules of the kidney cortex (11). If two or more fluorophores are simultaneously used, color-switching experiments are required to exclude a label-dependent bias.

Injection of fluorescently labeled anti-mouse GR1 (bi-specific for Ly6C and Ly6G) antibodies in wild-type (11) or CX3CR1-GFP^+/−^ mice (20) helps to discriminate classical monocytes and neutrophils from patrollers. Similarly, anti-mouse Ly-6C (clone HK1.4) can distinguish between classical (Ly6C^+^) and non-classical (Ly-6C^−^) monocytes in CX3CR1-GFP mice. GFP^−^ Ly-6C^+^ populations in the blood include neutrophils and some T cell subsets, whereas the GFP^+^ Ly6C^−^ subset unambiguously corresponds to patrolling monocytes in the blood. Labeling of CD115 (CSF-1R, clone AFS98), although highly specific for monocytes, is not recommended for intravital imaging, as it affects M-CSF signaling (30). To ensure that the imaged cells are located in the vessel lumen, a blood tracer (e.g., 70–200 kDa fluorophore-coupled dextran) must be coinjected. A gap in the tracer signal verifies the intraluminal position of the cell. In addition, fluorescently labeled anti-mouse CD31 (PECAM-1, clone 390) can be used to mark the endothelium. However, this antibody also labels neutrophils and platelets (31).

Together, neither genetic nor antibody labeling approaches alone achieve high specificity and sensitivity. It is therefore highly recommended to verify the validity of the labeling strategy using multiple methods.

## Imaging Platforms and Tissue Sites

Most vascular sites feature a unique molecular environment with tissue-specific patterns of intravascular leukocyte adhesion (31, 32). Therefore, the site of imaging should be carefully chosen according to the biological question. Due to the proximity of the vessels to the tissue surface, many tissues and organs are accessible for intravital upright confocal microscopy, which enables a penetration depth of about 100 µm. This includes the ear dermis, the mesentery, the cremaster muscle, the femoral and popliteal vasculature, the spleen, and the liver. While patrolling also occurs in the microcirculation of the kidney cortex, glomeruli as main functional units cannot be assessed in their entirety using confocal imaging. For these denser and highly scattering tissues, multiphoton microscopy is the preferred imaging modality (33). Patrolling, in contrast to rolling, describes a slow motion. Therefore, acquisition speeds of 1–0.5 frames/s are sufficient to describe the kinetics of patrolling. Tiled acquisition is possible. Modern 20–25× water immersion objectives with a NA around 1.0 offer reasonably high spatial resolution and a large field of view.

A challenge to intravital imaging is the intrinsic movement of tissues due to muscle twitching, peristalsis, and cardiac and respiratory cycles, which can strongly bias kinetic readouts. Tracheal intubation helps to reduce respiration-related motions. A respirator can be used with a pause at the plateau after inspiration or expiration. The muscular tone controlled by the autonomic nervous system can be suppressed by muscle relaxants. Restraining devices can be helpful in stabilizing the target tissue, yet require proper controls to rule out artifacts that may be introduced by the immobilization apparatus. For example, the widely used stabilization device with a suction chamber applies a vacuum (34) that can trigger trauma-induced neutrophil accumulation. Similarly, physical restrainers of vessels (35) directly impact on the adventitia and physiological flow conditions, and indirectly on endothelial cell biology (36, 37).

We recently developed an intravital live cell triggered imaging system for stable 2D and 3D two-photon imaging of large arteries that does not require physical restraint (33). This technology enables high-resolution video acquisition of leukocyte cell migration in the intravascular and intramural compartment of healthy and diseased arteries. The system has been optimized for the Leica platform, but can be mounted on any multiphoton microscope with external trigger control. It requires a trigger-box, non-invasive pulse oximetry, and a custom-made Arduino circuit with a Matlab-based software module to coordinate the pulse signal and frame acquisition. The system is versatile and can be used to study intra- and extravascular leukocyte behavior in many diseases, including atherosclerosis, renal artery stenosis, and vasculitis of large arteries.

## Kinetic Analysis of Patrolling

Blood-borne leukocytes interact with the endothelium in an orchestrated manner to leave the blood stream and exert their function in the surrounding tissue (31, 38). Members of the selectin and integrin families as well as cytokine receptors are sequentially engaging, resulting in capture, rolling, arrest, and extravasation. Although this process, referred to as the leukocyte adhesion cascade, differs qualitatively and quantitatively among different vascular beds and environmental signals, common key patterns have emerged (31, 38). As the leukocyte adhesion cascade consists of distinct migration steps, kinetic analysis of patrolling cells aids to delineate underlying molecular processes. These are summarized in Table 1.

**Table 1:** Microkinetic parameters to describe patrolling.

	Start	End	Unit	Description
Displacement	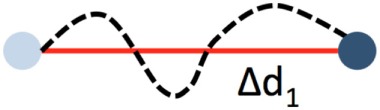		ΔXY_end_-XY_start_ in μm	Beeline traveled within a time period (e.g., 1 min)

Path length	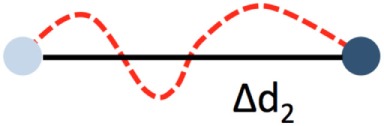		Path length in µm	Strongly affected by motion artifacts. False measurements also affect the confinement ratio

Duration	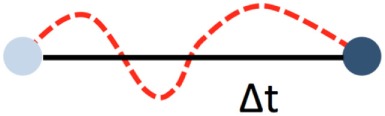		Total patrolling time in seconds	Depends on recording time. A minimum recording time of about 30 min is required in venules, and about 20 min in arteries

Velocity	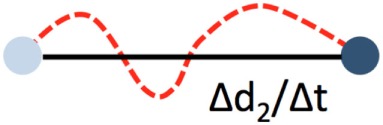		Path length per time in µm/min	Minimum time ideally >10 min. Jerky patrolling can be plotted as velocity over time

Confinement ratio, straightness	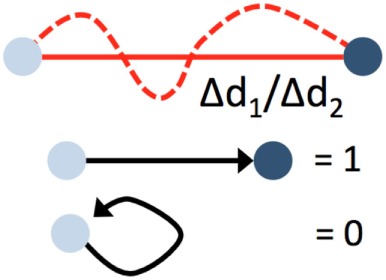		Displacement/path length. No unit	Straightness of the path. 1 = straight path. 0 = start and end position overlap

Path length and displacement describe the total (circuitous) path length and the direct distance (beeline), respectively. Confinement ratio is defined as the ratio of path length and displacement. A value of 1 signifies a straight path, and a value close to 0 a meandering/circular motion. Importantly, these parameters can change as a function of the length of the video recording, which therefore needs to be standardized and noted in the method sections. A recording time of 30 min has been found to be sufficient in most circumstances. Under inflammatory conditions, some patrollers show longer durations of interactions, which may necessitate longer recordings. Velocity is calculated as distance traveled over time and expressed as µm/min. While the velocity in a patrolling population in healthy vessels is mostly homogenous, disease conditions can provoke irregular patterns, e.g., in atherosclerotic arteries (18), that can be plotted as velocity over time. The metric “dwell time” has been used to describe short static phases, particularly in glomerular capillaries (39). Since patrolling can occur with or against the blood flow, the flow bias is an insightful parameter. By aligning all start points of all tracks, the dominant patrolling direction can be plotted (e.g., as tracks or rose plot). Numbers of active patrollers per vessel segment need to be normalized to vessel surface area visible in the intravital recording to account for out-of-focus segments or disease-related vessel perturbations (such as atherosclerotic plaque). To determine these parameters, several manual and automatic tracking tools are available in Fiji (ImageJ) (40) or Imaris (Bitplane).

Motion artifacts of intravital recordings can significantly affect kinetic measurements. Non-linear and linear transformations (translation, rigid body, affine, or scaled rotation) can be corrected during post-processing. If the automated tracking algorithm (e.g., using the centroid of the cell) works to more precision than the image resolution, an artificial sub-pixel back-and-forth motion will occur, resulting in a systematic overestimation of the path length and underestimation of the confinement ratio. Noise filters that remove sub-pixel movements smaller than the image resolution can remedy this issue.

The lack of highly specific reporter models and the difficulty of precisely distinguishing patrolling from other steps of the adhesion cascade poses challenges for data analysis. Criteria for the identification of patrollers include stable patrolling for 60 s or longer. In arteries, a 90-s threshold is recommended to safely discriminate motion artifacts and slow rolling from active patrolling. Patrolling velocity in microvessels and arteries is about 12 and 36 µm/min, respectively (11, 18, 20). To discriminate rolling from patrolling, a velocity threshold of 2 standard deviations (SDs) below the mean rolling velocity should be applied. Since the selectin requirements of non-classical monocyte rolling have not been studied, a clear definition of monocyte rolling before patrolling is not yet available.

**Figure 2:**
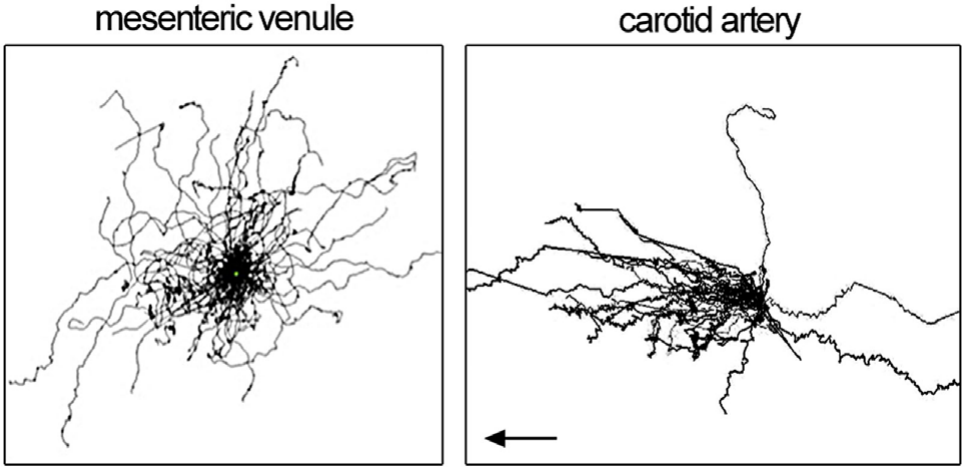
Patrolling tracks with centered start coordinates as spider pot. Each line represents one patrolling monocyte. Data show patrolling in mesenteric venules (left) and carotid artery (right panel). Left panel is adopted from Carlin et al. (11) and right panel from Quintar et al. (18). Flow direction in the left panel is not available.

Blood flow imposes directional shear forces on intravascular leukocytes. In the dermal, mesenteric, and kidney microcirculation, intravascular patrolling occurs mostly independent of the blood flow (migration regardless of flow direction). However, in arteries, a strong downstream flow bias has been detected (preferential migration with flow direction; Figure 2). The velocity and meandering migration paths are currently the only kinetic parameters that identify patrolling monocytes throughout the circulation.

## Tissue-Specific Molecular Requirements

Molecular requirements of patrolling are both site- and stimulus-specific with regard to adhesion receptors. Table S1 in Supplementary Material highlights the main findings currently available in the literature.

In homeostatic conditions, arterioles, capillaries, and postcapillary venules are populated by patrollers (11, 19, 20, 41). Several investigations in mice highlighted the critical role of integrins. Blockade of the leukocyte integrin LFA-1 (α_L_β_2_) results in immediate detachment of patrolling monocytes in all healthy tissues studied (19). In most cases, this leads to an increase of blood-borne non-classical monocytes as measured by flow cytometry, suggesting that around one-third of the marginal pool of non-classical monocytes is constantly engaged in vascular patrolling (19). Integrin α_L_ knockout mice also showed abolished patrolling although this global knockout does not allow unambiguous conclusions. Patrolling is reduced by around 50% in *ICAM-1*^−/−^ mice, and additional knockout of ICAM-2 completely eliminates patrolling (11). *ICAM-2*^−/−^ alone does not affect patrolling, suggesting that ICAM-1 is the major endothelial ligand for LFA-1 in patrolling monocytes, and ICAM-2 is a redundant binding partner (11). CD11b (integrin α_M_, Mac-1) inhibition does not reduce the numbers of patrollers in steady state venules of the ear dermis (19) and glomerular capillaries (41) but decreases dwell time and path length in the latter (41). Endothelial CCN1/CYR61 as a potential CD11b ligand is required for effective patrolling in mesenteric venules (20). Under homeostatic conditions, the chemokine receptor CX3CR1 is irrelevant in most vessels but not in uninflamed glomerular capillaries of the kidney (41). Treatment with pertussis toxin, a potent inhibitor of G_αi_ signaling required for integrin activation, does not affect patrolling in steady state mesenteric venules (11). Intravital microscopy of the ear dermis after adoptive transfer of human monocytes (5) and flow chamber experiments on human umbilical vein endothelium (HUVEC) (17) confirmed the role of integrin LFA-1 in human CD14^dim^CD16^+^ monocyte crawling on uninflamed tissue. Furthermore, blocking of CX_3_CL1 or VEGFR2 intensified patrolling via unknown mechanisms *in vitro* (17). These data show that LFA-1 integrin is mandatory for patrolling in all conditions, whereas the role of Mac-1, ICAM-1/2, and the CX3CR1 chemokine receptor varies.

Capillaries of the kidney glomerulus are a key target of renal inflammation and injury (42). Interestingly, many patrolling mechanisms seem to be different here. Using multiphoton intravital microscopy, about five non-classical monocytes were detected per hour in one glomerulus with an average dwell time of about 15–20 min (39, 41). Deficiency of CX3CR1 (using CX3CR1-GFP^+/+^ mice) or the combined blockade of β_2_ (CD18) and α_4_ (CD49d) integrins reduced the number of patrollers in uninflamed conditions (41). CD18 or α_4_ integrin inhibition alone does not have an effect (41). During the anti-glomerular basement membrane (GBM) antibody response (these antibodies trigger glomerulonephritis as in Goodpasture syndrome), primary adhesion of patrollers requires LFA-1, and the dwell time is reduced after CD11b blockade (41). During anti-GBM inflammation, CX3CR1 knockout mice showed changes in patroller recruitment and dwell times in a time-dependent manner (41). These data emphasize that adhesion requirements differ depending on the environmental and spatial context.

There are phenotypic similarities between monocyte patrolling and neutrophil “crawling.” The latter describes a slow, integrin Mac-1 (CD11b)-dependent meandering motion along the endothelium (43). P-selectin glycoprotein ligand 1 (PSGL-1) engagement, platelet interactions, and LFA-1-mediated arrest are mandatory for effective neutrophil crawling (43, 44). While both monocyte patrolling and neutrophil crawling include upstream and perpendicular motion and require endothelial ICAM-1 (43), only neutrophil crawling is known to necessitate an endothelial chemotactic gradient (45) and eventually results in site-directed extravasation (43). Moreover, in contrast to patrolling, crawling is evident only on activated endothelium. Of note, CXCR6^+^ NKT cells also show patrolling along liver sinusoids (46). Thus, neutrophil crawling and monocyte patrolling are two separate entities. Molecular pathways may be similar, but this remains to be investigated.

## Toll-Like Receptors (TLRs) and Patrolling

Patrolling has been studied in several mouse models of inflammation. TLRs act as pattern recognition receptors that monitor damage- and pathogen-associated molecular pattern molecules in the blood stream (47, 48). Direct application of TLR agonists on the vessel (“painting”) mounts a local response in a time-dependent manner (11, 18, 20), whereas systemic use is not suitable for imaging due to pan-endothelial activation (49). The painting results suggest that perivascular tissue-intrinsic mechanisms suffice to intensify patrolling. TLRs are highly expressed on non-classical monocytes (5, 11). The impact of direct TLR stimulation of monocytes has not been investigated using intravital microscopy.

Painting of the mesenteric vasculature with agonists for TLR2 (Pam3CSK), TLR3 [Poly(I:C)], TLR4 (LPS), or TLR5 (flagellin) induce an time-dependent increase in patrolling (50). An early increase after 30–60 min is seen after TLR2 and TLR9 activation, whereas TLR3 and TLR4 promote a late accumulation (around 3 h) (50). TLR2 and TLR9 are the strongest inducers in these experimental settings, leading to about 9- to 10-fold more patrolling monocytes after 3 h (50). An increase of patrolling upon TLR7-activation by R848 (Resiquimod) has been shown in the dermis (ear) (11, 19), the mesentery (19, 20), the kidney (11), and the carotid artery (18). Notably, R848 attracts patrollers to both the arterial (18) and venular (11) endothelium, suggesting a conserved endothelial response. In all tissues and across all TLR stimulants except for TLR9 agonists (50), patrolling becomes more intense and meticulous after stimulation, as evidenced by longer dwell times (reduced velocity), longer tracks, and lower confinement ratios. In contrast to homeostatic conditions, blockade of G_αi_ signaling by pertussis toxin, Mac-1 (CD11b) by antibody inhibition or CX3CR1-deficiency impede the upregulation of patrolling after TLR7 stimulation (11) (Table S1 in Supplementary Material). It thus seems that vascular activation by most TLRs suffices to intensify local surveillance by patrolling monocytes.

Patrolling monocytes can initiate a local neutrophil response *via* a TLR7-dependent paracrine secretion of pro-inflammatory cytokines, such as IL-1β, KC, TNF, CCL3, or IL-6 (11). Activated platelets are required to effectively ramp up patrolling and signal subsequent neutrophil recruitment in mesenteric vessels (20, 51–53). Interestingly, TLR3 and TLR4 agonists lead to early (30–60 min) neutrophil accumulation that is followed by patrolling monocytes, indicating a TLR-specific temporal response of leukocyte recruitment to the activated endothelium (50).

It remains unclear how “painting” of TLR agonists works. Several biological components could play a role. Pericytes can actively support abluminal leukocyte behavior in the subendothelial space (54, 55). Moreover, laminins as active constituents of the basement membrane affect the endothelial phenotype (56) and leukocyte extravasation (57). An active supply of adhesion receptors to the endothelial surface from the lateral border recycling compartment is another example how the endothelium can actively shape interaction with blood-borne leukocytes (58). The study of these functional units might shed light on the microenvironmental signals required for effective patrolling.

## Patrolling Kinetics in Venules and Arteries

In addition to venules (11, 19, 20), arterioles (4), and capillaries (11, 41), it was recently demonstrated in mice that the healthy arterial endothelium of large arteries is also monitored by patrolling monocytes (18). Hence, patrolling seems to be a universal surveillance mechanism throughout the circulation. However, molecular and biophysical conditions differ in microvessels and macrovessels and between venous and arterial endothelium (59). The vascular wall shear stress is low in venules and high in small precapillary arterioles (37). Due to their large circumference, large arteries have an intermediate shear stress profile (60, 61), which impacts on the functional phenotype (62). Moreover, different gene expression patterns between endothelial cells of the venular and arterial tree determine many differences in the molecular landscape involved in the leukocyte adhesion cascade (59). The details of monocyte patrolling in arteries, veins, arterioles, venules, and capillaries remain to be explored.

In homeostatic conditions, patrolling occurs at a velocity of 9 µm/min in venules of the kidney cortex (11), 17 µm/min in the dermal microcirculation (19), and about 36 µm/min in carotid arteries (18) (Table 2). Only in arteries, a clear downstream bias of patrolling was found (in direction of the flow; Figure 2). The dermal and mesenteric circulation as well as *in vitro* patrolling on HUVEC cells (17) showed hairpins (straight tracks with one sharp turn), loops, waves (meandering), and mixed forms (19). In arteries, predominantly the wave pattern was observed, whereas others only rarely occurred (18). The confinement ratio was determined at 0.6, 0.5, and 0.2 for kidney cortex venules, mesenteric venules, and the carotid artery, respectively. It is possible that the arterial confinement ratio is somewhat underestimated due to uncompensated motion artifacts (overestimation of the total path length). These observations point to an active role of shear forces and the position in the vascular tree on the agility of patrollers. Similarly, other leukocytes show cell type-specific reactions to shear forces. T cells preferentially migrate against the flow over short distances (63–65), whereas neutrophils show a downstream flow bias (64, 66). Kinetic measurements of patrolling monocytes in different vascular networks are summarized in Table 2.

**Table 2:** Microkinetic analyses of patrolling monocytes in different vascular beds.

		Carotid artery (18)		Dermal venules (19)	Kidney cortex (11)		Lung (12)	Mesenteric venules (20)
		Basal	R848	Basal	Basal	R848	Basal	Basal
Velocity	μm/min	36	19	17	≈9	≈7.5	10.2 ± 0.3	≈9
Duration	s	284	343	14	≈540	≈1,300	nd	≈1,200
Length	μm	134	124	249	≈80	≈150	nd	≈200
Confinement ratio		0.22	0.10	0.63	≈0.6	≈0.3	nd	≈0.55
Displacement	μm	31	12	162	≈28	≈41	nd	nd

*nd, not determined*.

Besides flow conditions, differing repertoires of endothelial adhesion receptors between venules and arteries could also account for differences in observed kinetics. This is supported by the finding of a differential requirement for the integrins LFA-1 and VLA-4 (α_4_β_1_) in arteries. VLA-4 blockade in R848-treated arteries alone is not effective (18). Blocking LFA-1 reduces patrollers by 50%, and sequential blockade of VLA-4 leads to a further 25% reduction (18). In contrast, LFA-1 blockade alone in R848-treated dermal or kidney cortex venules suffices to abolish patrolling completely (11, 19). A similar observation was made in uninflamed glomulerula of the kidney (41). In all tissues studied, stimulation with TLR agonists leads to a significant decrease of the confinement ratio, pointing to a higher dwell time (Table S1 in Supplementary Material). These data emphasize that large arteries are unique entities with regard to monocyte patrolling.

Analysis of integrin requirements in arteries compared to venules suggest site-specific mechanisms (18). However, monocyte heterogeneity (67) within the non-classical subset with subset specific vascular tropism could also contribute to this phenomenon. This possibility has not been sufficiently studied so far. The intermediate subset in humans (CD14^+^CD16^+^) has not yet been described in mice. The function of the MHC-II^+^ subset of non-classical monocytes remains unclear. New multiplexed single-cell technologies will help to classify human and mouse monocyte patrollers with high resolution.

## Patrolling in Atherosclerotic Arteries

Monocyte recruitment to the neointima is a disease-defining process in atherogenesis (2, 16, 68). It has been shown that monocyte rolling on explanted atherosclerotic endothelium is mostly P-selectin dependent (69), and adhesion is driven by VCAM-1 and its ligand integrin α_4_β_1_ (70, 71). Endothelial ICAM-1 and VCAM-1 expression is upregulated at lesion sites (72, 73). Genetic depletion or blockade of VCAM-1 leads to reduced plaque buildup (74). However, the concept of classical and non-classical monocyte subsets and their distinct functions was unknown at the time of these studies. While classical monocytes adhere early to plaque-prone endothelium, extravasate, and contribute to the lesional macrophage population (F4/80^+^ Ly6C^−^ I-A^b+^ phenotype) (75–77), far less is known about non-classical monocytes.

Western diet is known to trigger monocytosis and is thought to mainly affect the classical monocyte population (75, 77). Advances in intravital imaging have allowed to quantify the intravascular accumulation of non-classical monocytes in murine carotid arteries (18, 33). Wild-type C57Bl/6J mice fed western diet for 4–6 weeks and *apoE*^−/−^ mice fed western diet showed an 8- and 22-fold increase in the number of patrolling monocytes on the arterial endothelium, respectively (Video S1 in Supplementary Material) (18). Thus, it is reasonable to hypothesize that a concurrent relocation of non-classical monocytes to atherogenic endothelia throughout the body results in a pseudo-reduction of these cells in the blood. This leads to a systematic bias in the analysis of blood-borne monocytes.

How does patrolling take place in large arteries? Intravital imaging showed that monocytes can directly interact with the endothelium from free flow (18, 35), possibly with the help of platelets (78). Kinetic analyses suggest that patrolling is preceded by arrest (18), suggesting an integrin-dependent adhesion step before patrolling. While most non-classical monocytes showed patrolling behavior (40%) in plaque-prone arteries, some were also arrested (15%), rolling (20%), or showed mixed phenotypes (18). The latter includes cells with alternating patterns of patrolling and fast rolling (>60 μm/s). Patrolling velocity (33 vs. 21 µm/s) and confinement ratio (0.2 vs. 0.05) in plaque vicinity is significantly decreased compared to plaque distant sites (18), indicating that local endothelial cues can trigger meticulous patrolling. Mean duration of patrolling was observed between 4 and 7 min in atherosclerotic conditions (18). However, many cells engaged only in short phases of patrolling with subsequent fast rolling, followed again by slow patrolling (18). This observation points to intermittent engagement of selectin receptors that enable fast leukocyte rolling (38). A viable candidate for capturing and rolling is PSGL-1 (ligand for endothelial P-selectin and E-selectin). PSGL-1 is expressed in non-classical and classical monocytes (79). Of note, differential adhesion receptor requirements have been found in short- and long-term patrolling of human CD14^dim^CD16^+^ human monocytes *in vitro* (17). Detailed insights on heterogeneity and spatial arrangements of the endothelial receptor landscape affecting monocyte patrolling and rolling are currently unavailable.

What is the fate and function of plaque-patrolling monocytes? Most patrollers in plaque-prone arteries detach eventually and are carried away in the circulation. While patrollers can arrest and extravasate under certain inflammatory conditions have been described (19, 80), the extent and relevance of this pathway during atherogenesis is still debated. Further studies are required to establish the identity, the migratory route, and the phenotype of these cells in atherosclerosis.

Recent data suggest an endothelial protective effect of patrolling monocytes in early atherogenesis (18). Nr4a1^−/−^ *Apoe*^−/−^ mice on western diet develop aggravated atherosclerosis (81, 82), and it has been proposed that Nr4a1-deficiency causes hyper-inflammatory lesional macrophages (82). However, an additional explanation could be that patrolling monocytes confer early endothelial protection during hyperlipidemia. In this line, patroller-deficient Nr4a1^−/−^ mice show increased endothelial damage compared to wild-type controls as assessed by electron microscopy (18). Pleiotropic effects of Nr4a1-deficiency impede unambiguous conclusions.

To overcome these problems, a non-classical monocyte-specific knockout mouse was recently developed by excising the E2 superenhancer region upstream of the Nr4a1 promoter (83). In this mouse model, macrophages retain normal levels of activation during inflammatory conditions. The non-classical monocyte population is completely ablated. *E2*^−/−^*Ldlr*^−/−^ (LDL receptor knockout to trigger atherosclerosis) bone marrow chimeric mice on high cholesterol diet developed increased plaques along the aortic root (84). As shown by intravital microscopy, even in the non-plaque-prone femoral vasculature, patrolling activity was elevated during Western diet feeding beginning within 1 day. This required CD36, one of the receptors for oxidized low-density lipoprotein (OxLDL). Western diet feeding or OxLDL binding to CD36 induced F-actin formation, in part through adapter protein DAP12 and a member of the Src family kinase (84). These data hint at a chronic diet-induced inflammatory phenotype of non-classical monocytes that leads to increased endothelial recruitment even at a distance to plaque development.

## Conclusion

Vascular housekeeping by patrolling monocytes is a crucial process required for endothelial homeostasis. Many disorders entailing vascular inflammation might trigger increased patrolling activity including chronic kidney disease (85, 86), tumors (87), HIV infection (88), myocardial infarction (89), atherosclerosis (18), and medium and large-vessel vasculitis (90). Targeting monocyte patrolling may be thus be a useful therapeutic approach. Recently, it was shown that non-classical monocytes in lung allografts are involved in acute graft rejection by mediating neutrophil recruitment (91). Adverse effects of non-classical monocyte have also been found in dendritic cell remodeling after spine injury (92). To exploit the effects of patrolling in clinical settings, ways to selectively increase or abolish the non-classical monocyte population need to be explored. Alternatively, or in addition, strategies to selectively manipulate patrolling behavior may become available.

There is a high interest in understanding the molecular foundation of patrolling in different parts of the vasculature. Many studies have contributed to a deeper understanding of the biological impact in health and disease. Yet, compared to the vast body of work on neutrophil and lymphocyte recruitment, a multitude of questions remains to be addressed. High-resolution intravital microscopy will be a key technology in this endeavor.

## Author Contributions

KB researched the published papers and wrote a draft of the review. KL revised the review and wrote parts. PM discussed data and wrote a section. CH reviewed the review.

## Conflict of Interest Statement

The authors declare that the research was conducted in the absence of any commercial or financial relationships that could be construed as a potential conflict of interest. The reviewer SM and handling Editor declared their shared affiliation.

## Supplementary Material

Video S1 ILTIS intravital microscopy of the unrestrained carotid artery in CX3CR1-GFP/ApoE-KO mice at a heart rate of about 300/min. Collagen is blue (second harmonics), and the blood red (Dextran). Blood flow right to left.
