# Supplementary material for: Patrolling Mechanics of Non-Classical Monocytes in Vascular Inflammation
Source: Front Cardiovasc Med. 2017 Dec 19;4:80. doi: 10.3389/fcvm.2017.00080 (PMC5742122; doi:10.3389/fcvm.2017.00080)
Supplement: Supplementary file 1 [file table_1.docx]

***Supplemental table II:*** *Synopsis of published data on molecular requirements of patrolling in different tissues and conditions using intravital microscopy. The metric indicated under “observation” is the number of patrollers unless otherwise indicated. Clone is indicated for each antibody. PT = pertussis toxin. α-GBM = anti-glomerular basement membrane antibodies.*

| **cells** | **organ, vessel** | **label** | **condition** | **observation** | **reference** |
| --- | --- | --- | --- | --- | --- |
| mouse  Ly6C- | mesentery venules | CD11b+, GR1- | ICAM-1-/- | 50% decrease  (vs. wildtype) | Carlin ^11^ |
|  |  |  | ICAM-2-/- | no change  (vs. wildtype) |  |
|  |  |  | ICAM-1/2-/- | 98% decrease  (vs. wildtype) |  |
|  |  |  | Itgal-/- | 99% decrease  (vs. wildtype) |  |
|  |  |  | CCR2-/- | no change  (vs. wildtype) |  |
|  |  |  | Nr4a1-/- | 90% decrease  (vs. wildtype) |  |
|  |  | CX3CR1-GFP+/- | 100 μg PT for 2h | no change  (vs. untreated) |  |
|  |  | CX3CR1-GFP+/+ | CX3CR1-/- | no change  (vs. CX3CR1-GFP+/-) |  |
|  | kidney cortex venules | CX3CR1-GFP+/- | R848 200μg i.v. | slight increase  (vs. untreated) |  |
|  |  |  | R848 200μg local | strong increase  (vs. untreated, time dependent) |  |
|  |  |  | R848 200μg local, TLR7-/- | decrease (vs. R848 TLR7+/+) |  |
|  |  |  | R848 200μg local, PT 50μg | decrease (vs. R848 alone) |  |
|  |  |  | R848 200μg local, α-CD11b  (M1/70) | decrease (vs. R848 alone) |  |
|  |  | CX3CR1-GFP+/+ | R848 200μg local,  ­CX3CR1-/- | decrease (vs. CX3CR1-GFP+/- R848) |  |
| mouse  Ly6C- | ear dermis venules | CX3CR1-GFP-/+ Rag2-/- γc-/- | α-CD18  (GAME-46) | 98% decrease  (vs. untreated) | Auffray ^19^ |
|  |  |  | α-CD11b  (M1/70) | no change  (vs. untreated) |  |
|  |  |  | α-CD11a  (M17/4) | 99% decrease  (vs. untreated) |  |
| mouse  Ly6C- | carotid artery | CX3CR1-GFP+/- | western diet  (4-6 weeks) | 9x increase  (vs. healthy) | Quintar ^18^ |
|  |  | CX3CR1-GFP+/-  apoE-/- | chow diet | 8x increase  (vs. healthy) |  |
|  |  | CX3CR1-GFP+/-  apoE-/- | western diet  (4-6 weeks) | 22x increase  (vs. healthy) |  |
|  |  | CX3CR1-GFP+/+  apoE-/- | CX3CR1-/- | no change  (vs. CX3CR1-GFP+/- apoE-/-) |  |
|  |  | CX3CR1-GFP+/- | R848 local 5h | 8x increase  (vs. untreated, time dependent) |  |
|  |  |  | R848 local 5h, α-CD49d 40μg (PS/2) | no change  (vs. R848) |  |
|  |  |  | R848 local 5h, α-LFA1 50μg | 50% decrease  (vs. R848) |  |
|  |  |  | R848 local 5h, α-CD49d 40 μg (PS/2), α-LFA1 50 μg (TIB 217) | 75% decrease  (vs. R848) |  |
| human CD16+ CD14- | ear dermis venules | stained with Vybrant-DiD, transferred in  Rag2-/- Il2rg-/- CX3CR1-GFP+/- | α-LFA1 (clone 38) | 100% decrease  (vs. untreated) | Cros ^5^ |
| mouse  Ly6C- | mesentery venules | CX3CR1-GFP+/- | R848 100μg local 3h | 3x increase  (versus untreated, time dependent) | Imhof ^20^ |
|  |  |  | R848 100μg local 3h, α-CCN1 (polyclonal) 50μg | decrease  (vs. R848) |  |
|  |  |  | R848 100μg local,  platelet depletion | decrease  (vs. R848) |  |
|  |  |  | α-CD11b 50μg  (M1/70) | no change in numbers, decrease in velocity and path length  (vs. untreated) |  |
|  |  | CX3CR1-GFP+/+ | CX3CR1-/-,  R848 100μg local 3h | decrease  (vs. CX3CR1-GFP+/- R848) |  |
| mouse  Ly6C- | mesentery venules | CX3CR1-GFP+/- | R848 100μg local 3h | 3x increase  (vs. untreated) | Imhof ^50^ |
|  |  |  | Pam3CSK4 100μg local 3h | 10x increase  (vs. untreated) |  |
|  |  |  | poly(I:C) 100μg local 3h | 2x increase  (vs. untreated) |  |
|  |  |  | LPS 100μg local 3h | 1.5x increase  (vs. untreated) |  |
|  |  |  | flagellin 100μg local 3h | 2x increase  (vs. untreated) |  |
|  |  |  | CpG ODN 100μg local 3h | 9x increase  (vs. untreated) |  |
| mouse Ly6C- | kidney glomerular capillaries | CX3CR1-GFP+/+ | CX3CR1-/- | > 50% decrease, also decrease in dwell time  (vs. CX3CR1-GFP+/-) | Finsterbusch ^41^ |
|  |  | CX3CR1-GFP+/- | α-CD18  (GAME-46) | no change  (vs. untreated) |  |
|  |  |  | α-CD49d  (PS/2) | no change  (vs. untreated) |  |
|  |  |  | α-CD18, α-CD49d (GAME-46, PS/2) | 50% decrease  (vs. untreated) |  |
|  |  |  | 1-2h α-GBM, α-CD11a (M17/4) | decrease  (vs. 1-2h α-GBM) |  |
|  |  |  | 1-2h α-GBM, α-CD11b (5C6) | no decrease in numbers, dwell time decrease  (vs. 1-2h α-GBM) |  |
|  |  | CX3CR1-GFP+/+ | 0-1h α-GBM, α-CD11a (M17/4) | significant decrease, no decrease in dwell time  (vs. CX3CR1-GFP+/- 0-1h α-GBM) |  |
|  |  |  | 1-2h α-GBM, α-CD11a (M17/4) | decrease not significant, significant decrease in dwell time  (vs. CX3CR1-GFP+/- 1-2h α-GBM) |  |
| Mouse Ly6C- | Femoral/ popliteal large vessels | CX3CR1-GFP+/- | Chow | sparse patrolling | Marcovecchio^59^ |
|  |  |  | western diet 4 weeks | 4x fold increase  (vs. chow diet) |  |
|  |  |  | 100ug OxLDL | increase within 10-20min |  |
|  |  | CX3CR1-GFP+/+ | western diet  4 weeks | no change  (vs. CX3CR1-GFP+/-) |  |
|  |  | CD36-/- | western diet  4 weeks | no change  (vs. chow diet) |  |
|  |  | MSR1-/- | western diet  4 weeks | no change  (vs. chow diet) |  |
|  |  | TLR-7-/- CX3CR1-GFP+/- | western diet  4 weeks | no change  (vs. chow diet) |  |
|  |  | DAP12-/- | western diet  4 weeks | decreased  (vs. wildtype) |  |
